# Identifying Lung Cancer Patients Suitable for Segmentectomy: A Brief Review

**DOI:** 10.3389/fsurg.2021.637441

**Published:** 2021-07-08

**Authors:** Chunguo Wang, Sikai Wu, Rongwei Zhang, Ke Jin, Yicheng Qian, Ning Mao, Yun Liu, Miao Zhang, Ke Zhang, Renfeng Wang, Gang Huang, Min Zhang, Baofu Chen, Jianfei Shen

**Affiliations:** ^1^Key Laboratory of Minimally Invasive Techniques & Rapid Rehabilitation of Digestive System Tumor of Zhejiang Province, Linhai, China; ^2^Department of Thoracic Surgery, Taizhou Hospital of Zhejiang Province Affiliated to Wenzhou Medical University, Linhai, China; ^3^Department of Emergency, Chinese and Western Combined Hospital of Taizhou Wenlin, Taizhou, China; ^4^Department of Cardiothoracic Surgery, Yongchuan Hospital of Chongqing Medical University, Chongqing, China; ^5^Department of Cardiothoracic Surgery, The First College of Clinical Medical Science, China Three Gorges University, Yichang, China; ^6^Department of Cardiothoracic Surgery, Yichang Central People's Hospital, Yichang, China; ^7^Department of Cardiothoracic Surgery, Xuzhou Central Hospital, Xuzhou, China; ^8^Department of Thoracic Surgery, Affiliated Hospital of Hebei University, Baoding, China; ^9^Basic Research Key Laboratory of General Surgery for Digital Medicine, Baoding, China; ^10^3D Image and 3D Printing Center, Affiliated Hospital of Hebei University, Baoding, China; ^11^Department of Thoracic Surgery, Xiamen Branch, Zhongshan Hospital, Fudan University, Xiamen, China; ^12^Department of ThoracicSurgery, The Third Hospital of Hebei Medical University, Shijiazhuang, China; ^13^Department of Cardiothoracic Surgery, The First Affiliated Hospital of Chongqing Medical University, Chongqing, China

**Keywords:** lung cancer, segmentectomy, thoracoscopy, indications, NSCLC

## Abstract

**Background:** In 1995, a clinical randomized controlled study (RCT) conducted by the Lung Cancer Study Group (LCSG) pointed out that the lobectomy was the gold standard for treating early lung cancer. However, with the development of technology, the results of several retrospective studies have shown that the efficacy of pulmonary segmentectomy is equivalent to that of lobectomy. Currently, it is still controversial whether segmental resection or lobectomy should be performed for early lung cancer. Thus, we aim to summarize the indications of segmentectomy.

**Methods:** To conduct the review, previous researches involving indications of segmentectomy were collected from the literature using Pubmed. These articles were published and accepted in English in the medical literature from 2013 to 2020. We have focused on segmentectomy and its indications.

**Results:** A total of 176 articles were retrieved from the Pubmed database, of which 31 articles included indications for segmentectomy. We summarized the relevant content, and the potential and prospect of segmentectomy for the treatment of lung cancer were emphasized.

**Conclusions:** These findings have a number of important implications for future practice. Pulmonary segmentectomy is a very vital surgical procedure for select patients with lung cancer, which provides a novel approach for the treatment of lung cancer and the survival of lung cancer patients.

## Introduction

Historically, segmentectomy has mainly been used as a palliative treatment for patients with cardiopulmonary insufficiency. In addition, the results of a randomized controlled trial (RCT) conducted by the Lung Cancer Study Group (LCSG) in 1995 had a profound influence on the last 20 years of the use of lung segmentectomy for early lung cancer. At that time, lobectomy was regarded as the primary treatment for early-stage NSCLC, since the lung cancer recurrence rate of patients with early-stage lung cancer had decreased significantly. However, the study compared patients who underwent lung segment or wedge resection with those who underwent lobectomy, and the maximal tumor diameter of the patients included was 3 cm. Thus, this study did not accurately evaluate the oncological efficacy of lobectomy and segmentectomy (1).

There are 2 types of sublobar resection. One is wedge resection and the other is segmental resection. The distinction between the 2 is that lung segmental resection requires the oncology standard of lobectomy, such as the anatomy of the separation of pulmonary segment veins, arteries, bronchi, and the better removal of lung parenchymal tissue. In addition, compared with pulmonary wedge-resection, it is adequate for pulmonary segmental resection to maintain the shape of the remanent lung. However, the prognosis of the malignant tumor patient is better with a lymph node dissection between the pulmonary segments. In some cases, high-risk patients, such as elderly patients with early lung cancer who have a poor respiratory function reserve and a history of lung resection surgery, the surgeon treats the lung cancer with a sublobar resection to cope with the unpredictable surgical risks as well as the probable long-term damage to the quality of life and respiratory function of the patient (2).

Recently, with the wide application of computed tomography (CT) lung carcinoma screening and the progress and application of video-assisted thoracic surgery (VATS), as well as the advent of the era of “precise medicine” and the opening of the field of “minimally invasive treatment,” more and more thought about the traditional standard method of treating lung cancer for early lung cancer has been brought to us the of: Is it possible to use segmentectomy for early lung cancer? A large amount of literature has reported the use of VATS segmentectomy to treat early lung carcinoma. These studies confirmed the dependability and feasibility of VATS in the treatment of early pulmonary carcinoma. Recently, thoracic surgery clinicians have launched a fierce debate on segmentectomy or lobectomy for early lung carcinoma (3).

In this article, we briefly summarize and recommend surgical indications for segmentectomy based on existing clinical data and clinical practice experience.

## Methods

**Figure 1:**
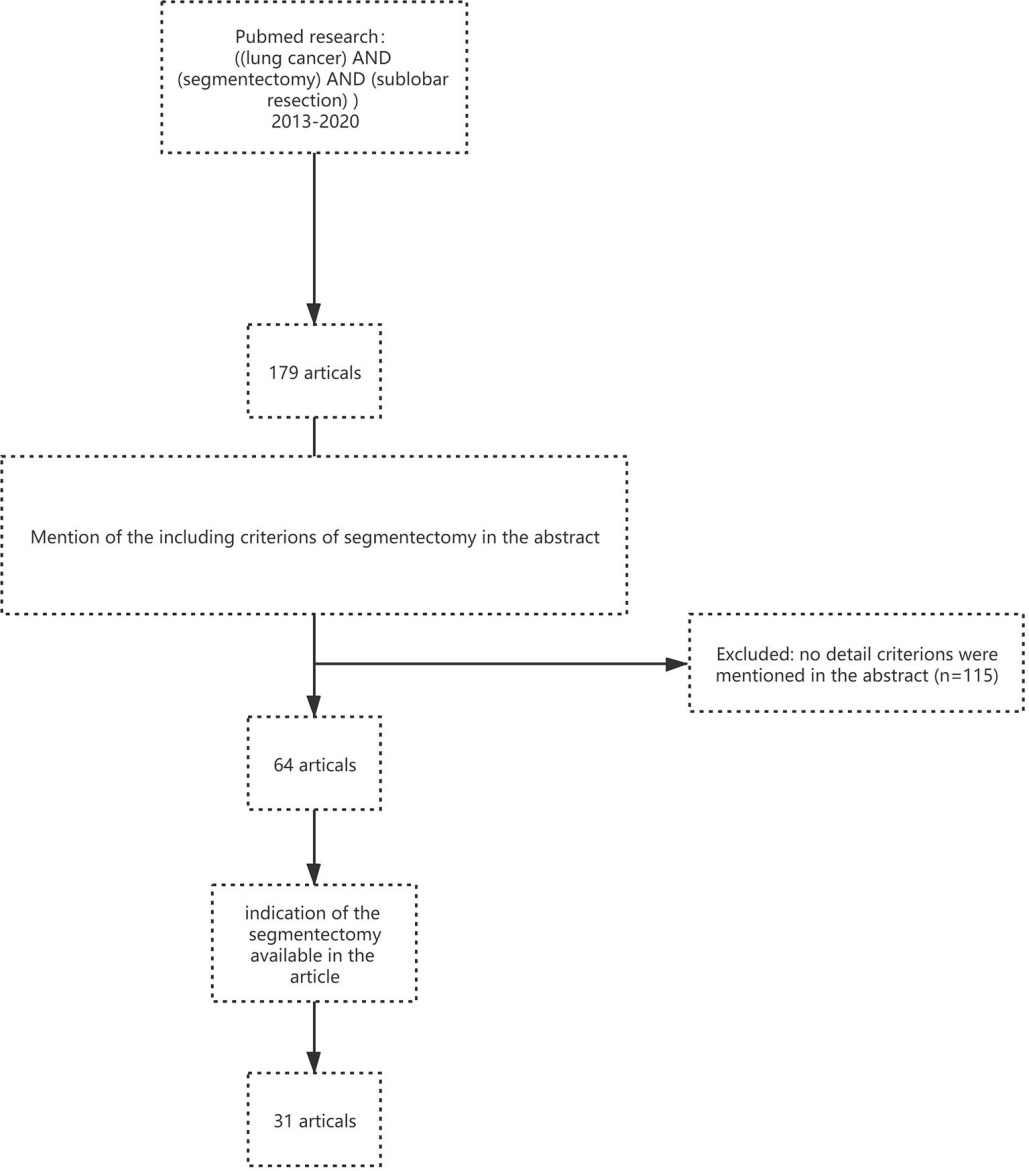
Flow chart of literature screening.

Here, we performed a comprehensive search via PUBMED using the following keywords: lung cancer; segmentectomy; sublobar resection; and indications to mainly review the surgical indications of segmental resection based on existing clinical data and clinical practice experience (Figure 1). These articles were published and accepted in English in the medical literature from 2013 to 2020. We summarized the indications for segmentectomy, and 31 relevant studies are included.

## Results

### Indications for Segmentectomy

#### Patients Aged ≥ 75 Years or Poor Pulmonary Function or Other Severe Comorbidities Who Cannot Tolerate Lobectomy

The comorbidities of pre-operation greatly influence a patient's prognosis. Sublobar resection may be a compromising surgical operation for elderly patients or patients with early stage pulmonary carcinoma, especially cStage I or II patients. Limited cardiopulmonary reserve as well as poor performance status were the primary indications for segmentectomy, sublobar resection or wedge resection (4, 5). A study by Perroni et al. (4) demonstrated that patients ≥75 years of age, with no regional lymph node disease, were more appropriate for segmentectomy. In addition, there was no difference in the survival of the more limited resection in those patients ≥71. Thus, sublobectomy remained an effective option in high-risk patients with limited pulmonary function, and pneumonectomy was a negative prognostic factor for long-term survival (6).

Moreover, a recent study by Yutaka et al. (7) demonstrated that the selection of segmentectomy was associated with older patients and those with poorer cardiopulmonary function. The procedure was proven to be relatively feasible, with no perioperative mortality, and provided a long-term survival that was comparable to lobectomy. Furthermore, the severity of the preoperative complications in these surgical subgroups had a more significant impact on long-term survival than the choice of surgical procedure.

Meanwhile, a study by Razi SS et al. (8) compared the survival rates among patients ≥75 years of age who underwent sublobectomy or lobectomy during the IANSCLC stage, the results revealed that in the N0 M0 NSCLC patients, the sublobectomy in the elderly was not inferior to lobectomy and should be considered a viable alternative in this high-risk population.

And in addition to preserving lung function, sublobectomy may also reduce the overall morbidity. For example, Kilic et al. reported that segmentectomy in patients over the age of 75 years resulted in a decrease in the incidence of major postoperative complications from 25.5% (lobectomy) to 11.5% (*P* = 0.02) (9).

At the same time, sublobectomy in patients in the T1aN0M0 stage of NSCLC may be better than lobectomy in the elderly and T1a stage NSCLC patients (tumor ≤ 2 cm). Survival in the patients with sublobectomy appeared to be comparable to that in the patients with lobectomy (2, 8). Furthermore, VATS segmentectomy could be performed safely and showed favorable 1-year survival. In addition, for patients with poor pulmonary function, it is significant to make decisions individually. In the meanwhile, we should consider the surgeon's experience, the size of the carcinoma, the location of the carcinoma, and the patient's preferences.

#### The Diameter of the Lung Cancer Lesion ≤ 2 cm

In the past 20 years, for patients with stage I NSCLCs ≤ 2 cm, the survival benefit of lobectomy over sublobectomy was reduced, with no significant difference in most contemporary cases (10).

Recent studies show that patients with T1N0 tumors ≤ 2 cm who underwent segmentectomy showed a 5-year overall survival rate that was comparable to standard lobotomy (4, 11, 12). According to the majority of the surgical guidelines, radical surgical excision of a tumor with a 2 cm margin as well as a tumor ≤ 2 cm is the accepted treatment (4, 13).

Following many previous studies, in the patients with tumors ≤ 20 mm, the results also reveal that the segmentectomy groups have a similar 5-year survival rate as the lobectomy groups (1, 7, 14).

In addition, recent research by Koike T indicated that compared with wedge resection, segmentectomy had a better prognosis, and for patients with stage IA NSCLC, they were inclined to select segmentectomy (15). Moreover, a previous study by Okada M that examined patients with cStage T1N0 peripheral tumors also reported that the patients who underwent sublobectomy had a similar mortality rate and lower perioperative lung morbidity than the lobectomy patients (14).

According to the research by Koike T, lobectomy and segmentectomy had a similar 10-year survival rate, which was 85% in patients with tumors ≤ 20 mm (16).

In addition, Raman V reported that the overall survival curves of patients undergoing segmentectomy was similar to lobectomy when the tumor size increased beyond 1 cm (17). Segmentectomy was not associated with a lower survival in a subgroup of 3,944 patients with tumors smaller than 15 mm, and a significant difference in survival was found in 32,676 patients with tumors >15 mm (17).

Similarly, Okada et al. studied 1,272 patients with stage I NSCLC who underwent sublobectomy or lobectomy and stratified them according to tumor size. The authors found no in lobular excision or lobe resection ≤ 2 cm, the 5-year cancer-specific survival was significantly different (8). Additionally, a study by Kates compared 688 patients who underwent sublobectomy, with 1,402 patients who underwent lobectomy for stage I NSCLC with sizes up to 1 cm. There was no statistically significant difference between overall and lung cancer-specific survival rates (9). Almost all the postoperative measurements for intraoperative and postoperative complications did not differ between segmentectomy and lobectomy, except that more air leakage was observed during segmentectomy (3).

Moreover, several studies have reported that for patients with early lung cancer (tumor ≤ 2.0 cm), the long-term survival was similar between the lung lobectomy groups and segmentectomy groups, which had 83.0–96.7% 5-year survival rates (18, 19).

A Surveillance Epidemiology End Results (SEER) database study demonstrated that in NSCLC patients (tumor 1 to 2 cm), segmentectomy has similar 5-year survival rates compared with lobectomy (20). This SEER database study indicated that in the early lung cancer, the patients who underwent sublobectomy had similar prognoses compared with the patients who performed lobectomy. However, in the intermediate group, the wedge resection had a poor outcome compared with lobectomy and segmentectomy. In the late lung cancer group, lobectomy showed a better prognosis than sublobectomy. This research represents a large review of the results after resection for T1a NSCLC (10, 12, 20).

Furthermore, in JCOG0201 study, it has demonstrated that the lung cancer of which the tumor size ≤ 2 cm, CTR (consolidation tumor ratio) ≤ 0.25 as well as the tumor size ≤ 3 cm, CTR ≤ 0.5 have a good prognosis in 5-year and 10-year overall survival. Therefore, segmentectomy may be a better option if the surgical margin is sufficient (21).

Moreover, based on the JCOG0201 study, the results of JCOG0804/WJOG4507L further demonstrated that for GGO dominant peripheral lung cancer, sublobar resection was suitable. And the study of JCOG0802/WJOG4506L have confirm that segmentectomy is the standard procedure in NSCLC patients with tumor ≤ 2 cm, CTR >0.5 (22).

#### The Proportion of Ground Glass-Like Composition in the Lung Cancer ≥ 50%

According to a recent study, with acceptable morbidity and mortality, VATS segmentectomy may be a treatment for CT1AN0M0 (ground-glass opacification (GGO) rate>50%) and is an acceptable option for NSCLC (19). Additionally, Terumoto Koike et al. showed that NSCLC patients with a higher GGO area on CT had better outcomes with sublobectomy (15).

A previous study by Sihoe AD indicated that the local recurrence rate after sublobectomy was over 17%, and after segmentectomy, it was only 2–8%. Meanwhile, predominantly ground-glass (non-solid) appearance on CT imaging could be a criterion for segmentectomy (9). In many studies, for patients with GGO lesions and micro-invasion adenocarcinoma, sublobectomy is considered acceptable (11).

In the meanwhile, a study by Yasuhiro Tsutani demonstrated that GGO-dominated tumors rarely showed pathologically aggressive features, such as lymphatic, vascular, or pleural infiltration, and lymph node metastasis. In addition, there was no significant difference in 3-year recurrence-free survival (RFS) among patients with GGO dominant tumors who underwent lobotomy (96.4%), segmentectomy (96.1%), and wedge resection (98.7%) (*P* = 0.44) (23).

One meta-analysis and two recent reviews of sublobar resection concluded that for minimal lesions or GGO lesions, sublobectomy, especially the AIS ≤ 2 cm, had a comparable overall survival rate and recurrence rate compared with lobectomy. Thus, sublobar resection is generally considered acceptable for adenocarcinomas with T1a tumors (11).

#### Imaging Confirmed the Doubling Time of the Tumor ≥400 d

Patients with fast-growing lung cancer (400 days) have a higher mortality rate than those with slow-growing lung cancer (400 days) or those with inert lung cancer (600 days) (4).

The National Comprehensive Cancer Network guidelines suggest that an anatomic segmentectomy is an option in some cases of early-stage peripheral pulmonary cancer. One of the criteria is that CT imaging confirmed that the doubling time of the tumor was ≥400 d (18). This criteria needs to be further identified, and a number of clinical studies are underway.

#### Pathology Confirmed Simple Adenocarcinoma *in situ* or Benign Lung Lesion

Due to the fact that minor lesions are inclined to represent an earlier lung cancer stage and a type of curable disease, many previous studies report that small tumors that undergo limited resection have better prognoses. The small tumors, with pathology confirmed simple adenocarcinoma *in situ*, are considered suitable for sublobectomy (16, 24).

A recent study revealed that the oncology effect of anatomic segmentectomy and wedge resection were equivalent, but this study was limited to patients with peripheral lung carcinoma ≤ 20 mm in size who are clinically lymph node-negative (5, 12, 25). According to the study by Sihoe AD, predictors of successful preoperative CT sublobar resection include the following: location in the outer third of lung parenchyma; lesions <3 cm; and no evidence of endobronchial involvement (9).

Wedge removal is not applicable for patients with a large range or deep anatomical locations of benign lesions or when the benign lumps are limited to a certain lung segment. In addition, if there is a history of solid malignant tumors and the intraoperative rapid freezing pathological result cannot confirm whether the nodule is the primary lung cancer or metastasis, the multiple pulmonary lesions need to be removed at the same time or may need to be removed in the future. If the pathological result of the lung lesion is suspicious metastasis or is difficult to confirm preoperatively, pulmonary wedge resection cannot be performed. Also, if it is located deep or adjacent to the blood vessels and bronchials, in order to avoid lobectomy, segmentectomy may be considered (9).

#### Complex Anatomical Segmentectomy in Lung Cancer

In our experience, single-level resection may not be sufficient to treat tumors that are located at the intersection of multiple levels, because the edge of the resection is not guaranteed and therefore the risk of recurrence may be increased (24).

A recent study demonstrated that complex anatomical segmental resection using traditional manual VATS is very challenging and more difficult than standard lobectomy. Thus, only a very large center can enable the surgeon to achieve appropriate surgical skills (4).

According to Macke RA, for the majority of patients, the observed mean 107 mL decrease in FEV1 after a 1–2 segmental resection is unlikely to be clinically significant, while the 286 mL decrease after a 3–5 segmental resection seems more likely to have adverse clinical effects, regrading overall function and quality of life (26).

**Figure 2:**
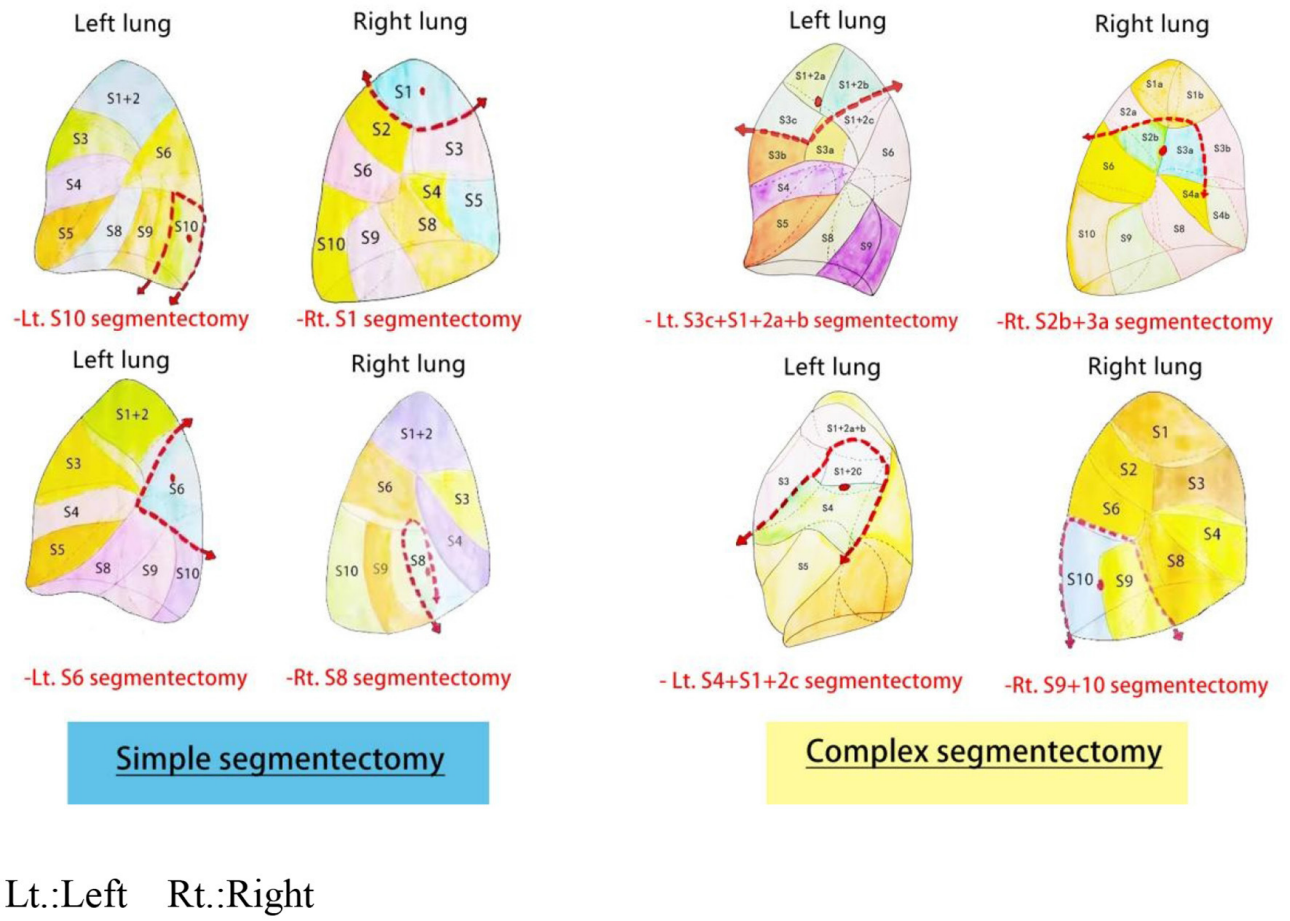
Part of the structure of the lung anatomic segmentectomy.

For nodules located at the margin of a segment or between adjacent segments, extensive segmentectomy is similar to an extended wedge resection: segmentectomy plus wedge resection (27). But, not all pulmonary nodules which meet this criterion are fit for combined subsegmentectomy. For instance, when the pulmonary nodules are located centrally and the distance between the bronchial origin of the subsegment in question and the nodules is no longer than the size of the nodules, combined subsegmentectomy does not identify a reliable surgical margin, while lobectomy is necessary (27). The part of the structure of the lung anatomic segmentectomy was shown in Figure 2.

## Discussions

Because of the increasing aging trend in China and the popularity of CT lung carcinoma screening, the number of aged patients with early lung carcinoma is increasing. Aged patients often have one or more systemic diseases, the most common being lung diseases, such as emphysema, chronic bronchitis, and even pulmonary heart disease. For these patients with different complications, the NCCN guidelines (non-small cell lung carcinoma) point out that sublobar resection, that is, pulmonary wedge resection or segmentectomy, can be used in technically permitted and some situations without increasing the risk of surgery. Moreover, we briefly summarized and recommended surgical indications for segmentectomy based on the existing clinical practice experience and clinical data.

In a recent study, we observed that there was no local intrathoracic recurrence for GGO-dominant tumors which underwent sublobar resection. However, due to the inertia of the GGO-dominant tumors, we should conduct a longer follow-up (4).

A study by Schuchter et al. demonstrated the results of 182 segmentectomy (thoracotomy and VATS) and 246 segmentectomy in NSCLC stage Ia and Ib patients, and the overall recurrence rates after segmentectomy (17.6%) and lobectomy (16.7%) were similar (24).

For patients with a small earlier tumor and with severe comorbidities, a wedge resection seems to be a better treatment option. However, for patients with a higher surgical risk, the benefit of segmental resection in the assessment of the lymph nodal and the margin of lung parenchyma should be considered (28).

On the basis of the previous guidelines, lobectomy plus systematic lymph node sampling or mediastinal lymph nodal dissection was the primary treatment for patients with the c-Stage IA NSCLC. Nonetheless, for patients with medicable surgically c-Stage IA NSCLC that have a worse lung reserve or other issues, sublobectomy seems to be a better choice. The patients cannot tolerate lobectomy, and thus, sublobectomy might be suitable (1).

However, up to now, for patients at high risk for cStage IA NSCLC, the efficacy of anatomic segmentectomy and wedge resection has not been clearly demonstrated. Compared with wedge-shaped resection, segmentectomy has a low local recurrence and mortality rate. Specifically, sublobar resections can reserve more lung parenchyma. Furthermore, the main finding of this report is that in patients with clinical stage cT1N0, wedge resection and anatomical segmentectomy may be equivalent to sublobectomy for the tumor (5, 15). A recent report showed that wedge resection and anatomic segmentation may be the tumor equivalent of sublobar resection in patients with clinical stage CT1N0 (5, 18).

According to previous research by Robert Dziedzic, the 3-year and 5-year overall survival rates between sublobectomy and lobectomy are not obviously different. Moreover, for patients with stage IA NSCLC, segmentectomy, rather than wedge-shaped resection, may be regarded as a replacement for lobectomy (29).

In the meanwhile, a previous study reported that preoperative comorbidities are an important prognostic factor. Thus, segmentectomy, which is a compromise surgical operation, might offer better outcomes for patients with complex preoperative comorbidities in c-stage IA NSCLC (7).

Compared with the surgical form and excision extension of segmentectomy, wedge resection is less invasive. In addition, according to trials in Japan, wedge resection is performed more often than segmentectomy. Therefore, for this high-risk population, wedge resection was identified as a standard surgical procedure. In a previous trial study, the definition of high risk included known risk factors in lung resection, such as forced expiratory volume in 1s (FEV1), diffusing capacity of the lung for carbon monoxide, pulmonary arterial hypertension, hypoxemia, hypercapnia, age, and poor left ventricular performance (1, 13).

Furthermore, we also discovered that compared with open surgery, minimally invasive surgeries, such as VATS, decrease the patient's complication or attack rate. However, the intuitiveness and the flexibility of this technique seem to be limited. Luckily, the introduction of robots has improved this situation and make sublobectomy safer and easier to conduct (4).

Moreover, a study by Mingming Ren shows that VATS segmentectomy was performed safely and is a method with favorable 1-year survival. It may be the ideal surgical procedure for patients with solitary pulmonary nodules in early-stage lung cancer, especially for those with limited cardiopulmonary reserve or significant comorbidities (27).

In addition, a study by Noriyoshi Sawabata demonstrated that sublobectomy of NSCLC provides favorable results in cases of R0 resection, which can be confirmed by cytological techniques with cells extracted from the margins, and an adequate margin distance/tumor size (M/T) ratio >1 (30).

For the high-risk patients, the effect of segmentectomy was better. Nevertheless, the 5-year OS and disease-free survival (DFS) of lobectomy seem to be higher than segmentectomies for tumors >20 mm. Most studies report that there is no obvious difference for the 5-year OS and DFS for tumors <20 mm (1).

An intraoperative assessment of lymph node metastasis and an adequate surgical margin may be a viable surgical approach for pure solid tumors of clinical T1A-BN0M0 lung cancer, and patients who undergo segmental resection of tumors in the right upper lobe or basal segment have a higher rate of regional recurrence. Thus, it is necessary to perform these procedures carefully (25).

Recently, a new technology, known as VAL-MAP2.0 technology, combines dye labeling and endobronchial placement of microcoils, making three-dimensional (3D) lung mapping possible. The accuracy and convenience of pulmonary resection are further improved with this technology (31).

In addition, under 3D navigation, sub-combined thoracoscopic combined sub-segmentectomy (CSS) was a safe operation for patients with intersegmental nodules, ensuring safe margins and sparing more pulmonary parenchyma to achieve anatomical resection. This will serve as a new technology to treat early lung cancer (27).

Furthermore, the JCOG1909 multi-institutional randomized phase III trial, which compares the clinical outcomes of wedge resection and segmentectomy, is underway, and the conclusion will be worth further discussion (13).

## Conclusions

In short, our study summarized the indications of segmentectomy. Accurately grasping the indications of segmentectomy can maximize the retention of healthy lung tissue, reduce the loss of lung function, and also ensure the safety and effectiveness of surgery. However, segmentectomy is a complex surgical option with high technical requirements, and thus, thoracic surgeons need to go through strict specialized training to conduct this surgery.

## Author Contributions

JS, CW, and SW searched the literature and drafted and revised the manuscript. RZ and SW made and processed images. KJ and YQ processed the date. BC and MZ revised and approved the final manuscript. All authors contributed to the article and approved the submitted version.

## Conflict of Interest

The authors declare that the research was conducted in the absence of any commercial or financial relationships that could be construed as a potential conflict of interest.
